# Idiopathic Pulmonary Fibrosis: A Comprehensive Review of Risk Factors, Genetics, Diagnosis, and Therapeutic Approaches

**DOI:** 10.3390/biomedicines14010090

**Published:** 2026-01-01

**Authors:** Lamiyae Senhaji, Nadia Senhaji, Meriame Abbassi, Mariem Karhate, Mounia Serraj, Mohammed El Biaze, Mohamed Chakib Benjelloun, Karim Ouldim, Laila Bouguenouch, Bouchra Amara

**Affiliations:** 1 Pulmonology Department, HASSAN II University Hospital Center, Fez 30050, Morocco; 2 Epidemiology and Research in Health Science Laboratory, Faculty of Medicine, Pharmacy, and Dental Medicine of Fez, Sidi Mohamed Ben Abdellah University, Fez 30050, Morocco; 3 Human Nutrition, Bioactive Compounds and Oncogenetics Research Team, Faculty of Sciences, Moulay Ismail University, Meknes 50000, Morocco; n.senhaji@umi.ac.ma; 4 Biomedical and Translational Research Laboratory, Faculty of Medicine, Pharmacy, and Dental Medicine of Fez, Sidi Mohamed Ben Abdellah University, Fez 30050, Morocco; 5 The Higher Institute of Nursing Professions and Health Techniques, Fez 30000, Morocco; 6 Department of Medical Genetics and Oncogenetics, HASSAN II University Hospital Center, Fez 30050, Morocco

**Keywords:** idiopathic pulmonary fibrosis, IPF, genetic, physiopathology, comorbidities, management

## Abstract

Idiopathic Pulmonary Fibrosis (IPF) is a severe, chronic, progressive lung disease classified within interstitial lung disorders. It predominantly affects individuals aged 50 to 70 years, with a prognosis of 3–5 years post-diagnosis. The pathophysiology of IPF is complex, involving an interplay of genetic predisposition, environmental exposures, and age-related factors. A significant genetic component is evident, with key contributions from rare variants in telomere maintenance genes (e.g., *TERT* and *TERC*) and surfactant protein genes (e.g., *SFTPA* and *SFTPC*), as well as a strong association with a common promoter variant in the *MUC5B* gene. The diagnosis is established through high-resolution computed tomography (HRCT) and, when necessary, histopathological analysis. The search for reliable biomarkers is a key area of research, with molecules such as KL-6, SP-A, SP-D, and MMP-7 showing potential for aiding in diagnosis, prognosis, and monitoring disease activity. While antifibrotic therapies (Pirfenidone and Nintedanib) have revolutionized management by slowing the decline in lung function, the therapeutic landscape continues to evolve. Ongoing research efforts are focused on integrating clinical, radiological, genetic, and biomarker data to facilitate early diagnosis and develop personalized treatment strategies to improve patient outcomes.

## 1. Introduction

Idiopathic Pulmonary Fibrosis (IPF) is a chronic, progressive fibrotic interstitial lung disease of unknown cause. The clinical burden of IPF is substantial, marked by a relentless decline in lung function, debilitating symptoms of progressive dyspnea and a cough, and a grim prognosis, with a median survival of only 3–5 years after diagnosis, which is worse than that of many cancers. While the advent of two approved antifibrotic therapies, Pirfenidone and Nintedanib, has been a critical step forward, they do not represent a cure. These treatments only slow the rate of functional decline and neither halt the disease nor reverse the established fibrosis. Their clinical utility can also be limited by considerable side effect profiles, and lung transplantation, the only definitive therapy, is an option for only a small, highly select group of patients. This landscape underscores a significant unmet therapeutic need for more effective and better-tolerated treatments. Therefore, elucidating the complex molecular and cellular mechanisms that drive the pathogenesis of IPF is an urgent priority, as it is fundamental to identifying novel therapeutic targets and developing transformative strategies for this devastating disease. This review provides a comprehensive overview of the current understanding of IPF pathogenesis, diagnostic approaches, and therapeutic management.

## 2. Epidemiology

Determining the epidemiology of IPF presents a real challenge given the variability in findings across multiple epidemiological studies. Most of these studies have been conducted in Europe and North America, with limited studies in Asia. Generally, IPF accounts for 20 to 50% of idiopathic ILD. The age of onset is the sixth to the seventh decade, with an mean age of 66 years and a notable male predominance (70%) [1]. This disease affects approximately 3 million individuals globally [2]. Its incidence ranges from three to nine cases per 100,000 individuals per year in North America and Europe, 1.2 to 3.8 per 100,000 people per year in Asia, and 0.4 to 1.2 per 100,000 people per year in South America [3]; while the prevalence is estimated between 10 and 60 cases per 100,000 individuals [4]. Notably, there are no studies focusing on the African population, and there are only two multi-ethnic studies, one conducted in Paris and the other in the United States. The first revealed that the prevalence of IPF is higher among North Africans compared to Europeans and Afro-Caribbeans (26.9 vs. 5.8 vs. 4.2 cases per 100,000 individuals) [5]; the second showed that IPF diagnosis was more frequent among African Americans, who also displayed an earlier mortality rate [6]. In Morocco, a cross-sectional survey involving nine reference hospital centers reported a total of 278 patients, estimating the prevalence and incidence of IPF to be at 1.26 and 0.6 cases per 100,000 inhabitants per year. The mean age was 61 years, again with a clear male predominance (75%) [7]. The mortality associated with IPF remains high, particularly among untreated patients, with the median survival estimated at 3.8 years [range: 3 to 5 years] [8].

## 3. Pathogenesis

### 3.1. The Central Role of Alveolar Epithelial Injury on Profibrotic Pathways

The pathogenesis of IPF remains incompletely understood, but it is believed to arise from the aberrant repair process of a complex interaction between genetic factors and environmental factors.

**Figure 1:**
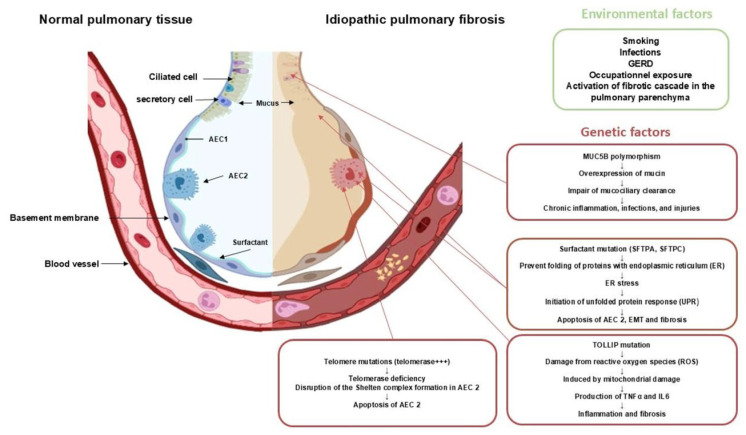
**The Role of genetic variants in initiating profibrotic processes.** This diagram illustrates how specific genetic mutations and polymorphisms contribute to the initial stages of IPF pathogenesis. Variants in telomere-related genes (e.g., *TERT* and *TERC*) lead to premature cellular senescence and an impaired regenerative capacity of alveolar epithelial cells. Mutations in surfactant protein genes (e.g., *SFTPA/C*) cause protein misfolding, leading to endoplasmic reticulum (ER) stress and apoptosis of type II pneumocytes. The common *MUC5B* promoter polymorphism results in mucociliary dysfunction, making the epithelium susceptible to injury. Variants in *TOLLIP* disrupt innate immunity, leading to an aberrant response to micro-injuries. Collectively, these genetic factors render the alveolar epithelium vulnerable, initiating a cascade of events beginning with epithelial injury and stress, setting the stage for the subsequent fibrotic response.

**Figure 2:**
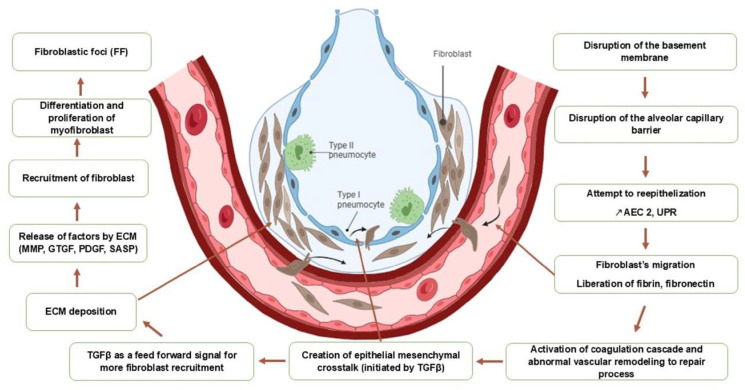
Schematic overview of the pathogenesis of Idiopathic Pulmonary Fibrosis. This figure depicts the aberrant wound healing process that drives fibrosis in IPF, following the initial triggers outlined in Figure 1. The process begins with the following: (1) Repetitive epithelial injury, caused by a combination of genetic susceptibility and environmental factors. This leads to (2) activation of TGF-β1, a potent profibrotic cytokine, which promotes apoptosis of epithelial cells and drives the epithelial–mesenchymal transition (EMT). TGF-β1 subsequently stimulates (3) fibroblast proliferation and differentiation, causing resident fibroblasts to proliferate and transform into myofibroblasts. These activated myofibroblasts are the primary source of extracellular matrix (ECM) components. Finally, this leads to (4) the formation of myofibroblast foci, which are dense accumulations of myofibroblasts and ECM that progressively remodel the lung architecture, leading to irreversible scarring, honeycomb cyst formation, and a loss of lung function.

Genetic abnormalities, in general, cause dysfunctions in protein production, which in turn disrupt alveolar cell behavior and induce apoptosis. This dysfunctional epithelium becomes vulnerable to microtrauma induced by environmental factors and is unable to regenerate. These microtraumas will be responsible for the destruction of the basement membrane and the alveolar capillary barrier, resulting in the leakage of proteins (fibrin and fibronectin) from the capillaries towards the interstitial and alveolar tissue, thus activating a cascade of coagulation and vascular remodeling attempting to repair this epithelium. Subsequently, there will be stimulation of fibroblasts leading to the production of profibrotic mediators, the most important of which is TGFβ1, responsible for promoting apoptosis of epithelial cells, facilitating epithelial–mesenchymal transition, and inducing the production of additional profibrotic mediators, ultimately resulting in the activation and transformation of fibroblasts into myofibroblasts. The accumulation of fibroblasts and myofibroblasts organize on the extracellular matrix, forming fibrotic foci. These fibrotic foci represent the starting point of progressive pulmonary remodeling, culminating in architectural distortion and the development of pulmonary fibrosis (Figure 1 and Figure 2) [4,9,10].

### 3.2. The Role of Epigenetics in Modulating Fibrosis

Epigenetics encompasses heritable changes in gene expression that occur without altering the DNA sequence, notably DNA methylation, histone modifications, and the regulation by non-coding RNAs. In Idiopathic Pulmonary Fibrosis (IPF), these mechanisms reprogram cellular phenotypes toward profibrotic, inflammatory, and maladaptive repair states, thereby shaping disease initiation and progression.

DNA methylation, mediated by DNA methyltransferases that add methyl groups to CpG-rich promoter regions, restricts transcription factor binding and compacts chromatin into a less accessible state. IPF lung fibroblasts exhibit genome-wide methylation alterations compared to the non-fibrotic controls, and pan-genomic integrative analyses have linked changes in CpG methylation to approximately one fifth of the corresponding differences in gene expression, revealing novel genes and pathways implicated in IPF pathogenesis [11,12]. These findings indicate that methylation actively contributes to profibrotic reprogramming and may yield actionable biomarkers and therapeutic targets.

Histone modifications further tune chromatin accessibility and transcriptional output through acetylation, methylation, phosphorylation, ubiquitination, and sumoylation. In IPF, the nuclear hyperactivity of acetyltransferase EP300 suppresses HDAC1 and disrupts the MiCEE ribonucleoprotein complex, perturbing higher-order genome organization. Pharmacologic or genetic inhibition of EP300 consistently attenuates fibrotic phenotypes across primary IPF fibroblasts in vitro, the bleomycin mouse model in vivo, and precision cut lung slices ex vivo, positioning EP300 as a compelling therapeutic target for dampening profibrotic transcriptional programs [13].

Non-coding RNAs add an additional regulatory layer. Altered microRNA profiles influence fibroproliferation, epithelial–mesenchymal transition, and TGF β1 signaling, thereby reinforcing fibrogenic circuits [14]. Long non-coding RNAs regulate genes in cis and trans by engaging chromatin remodeling factors and RNA binding proteins; multiple lncRNAs, including H19, PFAR (NONMMUT065582), CHRF, uc.77, AJ005396, and others, have been implicated in IPF development and progression [15]. Collectively, miRNAs and lncRNAs represent promising diagnostic and prognostic biomarkers, and provide avenues for RNA-based therapeutics, such as miRNA mimics or inhibitors and lncRNA-directed modulation [16].

## 4. Risk Factors

Several risk factors can explain the pathogenesis of the disease. These risk factors can be categorized into two main types:

### 4.1. Intrinsic Risk Factors

#### 4.1.1. Genetic Predisposition

Genetic factors play a crucial role in IPF susceptibility. These are broadly categorized into rare, high-penetrance variants and more common, low-penetrance polymorphisms. Rare mutations are often found in familial pulmonary fibrosis and typically involve genes critical for telomere length maintenance (e.g., TERT and TERC) or surfactant protein function (e.g., SFTPA1, SFTPA2, and SFTPC). These mutations can lead to premature cellular senescence and alveolar epithelial cell dysfunction, respectively. The most significant common variant is a polymorphism in the promoter region of the MUC5B gene (rs35705950), which is strongly associated with both familial and sporadic IPF by promoting mucociliary dysfunction. Other common variants in genes, such as TOLLIP, have also been linked to disease susceptibility and progression, highlighting the polygenic nature of IPF. These genetic factors are thought to render the lung epithelium vulnerable to injury, initiating the fibrotic cascade when combined with other risk factors like age and environmental exposures (Table 1).

#### 4.1.2. Age

IPF is often diagnosed in individuals aged between 50 and 70 years. Numerous studies indicate that both the incidence and prevalence of the disease increase with age. This can be attributed to cellular modifications occurring with age; the shortening of telomeres, the accumulations of genetic and epigenetic alterations, mitochondrial dysfunction, cellular senescence, and a decline in the differentiation capacity of stromal cells all can promote fibrosis [9,63,64].

#### 4.1.3. Male Gender

The prevalence of IPF is notably higher in men (70%). Initial hypotheses suggested that this disparity might be linked to higher rate of smoking and occupational exposure among men. However, research has demonstrated that even when women are exposed to similar risk factors, they exhibit a lower incidence of this disease. The potential influence of male sex hormones has been observed in murine models but requires further investigation in humans [9].

#### 4.1.4. Microbiome

Recent research has shifted focus to the lung microbiome, suggesting that alterations in the microbial communities of the lower respiratory tract may contribute to IPF pathogenesis. Studies have shown that patients with IPF exhibit an increased bacterial burden and a reduced microbial diversity—a state known as dysbiosis—compared to healthy controls, with certain bacterial genera like *Staphylococcus* and *Streptococcus* being more abundant [65]. It is hypothesized that this dysbiosis could perpetuate inflammation and fibrosis [66]. However, the evidence supporting a causal role for the microbiome is still emerging. Most studies are cross-sectional, making it difficult to determine if dysbiosis is a cause or a consequence of the established fibrotic environment [67]. Furthermore, results can be influenced by confounding factors such as patient medications, comorbidities, and disease severity. Technical challenges, including the potential for contamination during bronchoscopy and the low biomass of the distal lung, also complicate the interpretation of these findings [68]. Future longitudinal studies are needed to clarify the role of the lung microbiome in initiating or driving IPF progression.

#### 4.1.5. Gastroesophageal Reflux

An association between gastroesophageal reflux disease (GERD) and IPF is well-documented, with a high prevalence of abnormal acid reflux found in IPF patients, even in those who are asymptomatic. The leading hypothesis suggests that the recurrent microaspiration of gastric contents can cause chronic epithelial injury and activate profibrotic pathways in the lungs [69]. However, this association must be interpreted with caution, as a definitive causal link has not been established, and the relationship is considered complex and controversial. The relationship may be confounded by shared risk factors, and it remains debated whether GERD is a cause of lung fibrosis or a consequence of it. This ambiguity is further highlighted by clinical trials involving anti-acid therapies (e.g., proton pump inhibitors), which have yielded conflicting results and have not demonstrated a clear benefit in slowing IPF progression [70,71]. Therefore, while GERD is an important comorbidity to manage, its precise role in the pathogenesis of IPF remains an area of active investigation.

### 4.2. Extrinsic Risk Factors

#### 4.2.1. Smoking

Numerous studies have proven that smoking is the most significant risk factor associated with the development of IPF. Others indicate that smokers develop IPF at an earlier age compared to nonsmokers or former smokers, and they also exhibit a reduced mean survival rate relative to these groups [72].

#### 4.2.2. Environmental and/or Professional Exposure

Certain occupational or environmental exposures have been implicated in the etiology of IPF through various case–control studies. Notably, exposure to dust from wood, metal, textiles, stone, and agricultural environments (livestock) has been linked to the disease. Other less common exposures have also been described, including those related to hairdressing, bird breeding, dentistry, and living in urban or highly polluted environments.

## 5. Diagnosis

IPF typically manifests around the sixth decade of life, with a notable male predominance. The clinical signs are nonspecific, with patients commonly reporting progressively worsening dyspnea over months to years, associated with a chronic cough. Sometimes, patients may remain asymptomatic, with the condition being incidentally discovered during a chest CT scan conducted in another context.

Clinical examinations frequently reveal crackles and clubbing in approximately 25 to 50% of cases [1,73]. Some patients may exhibit signs of pulmonary hypertension (PH). A thorough medical history is essential, including inquiries about environmental or occupational exposure, medication use, or familial history of ILD or systemic symptoms, which are critical for the differential diagnosis [74].

Functionally, patients typically demonstrate a reduced forced vital capacity FVC and a diminished diffused lung of Carbone monoxide DLCO. However, in some cases, respiratory function may be normal at early stages or if coexisting with emphysema, where only the DLCO is low. A low FVC with a reduced DLCO and limited distance covered during the 6 min walk test are indicators of poor prognosis and elevated risk of mortality [75].

All patients who present with chronic clinical signs with a chest X-ray suggesting ILD should undergo a high-resolution chest CT, performed in breath-hold with deep inspiration and end expiratory cuts, without the injection of contrast, with thin cuts. This imaging allows for the identification of the patterns: definite Usual Interstitial Pneumonia (UIP), probable UIP, or indeterminate UIP [73,76].

**Figure 3:**
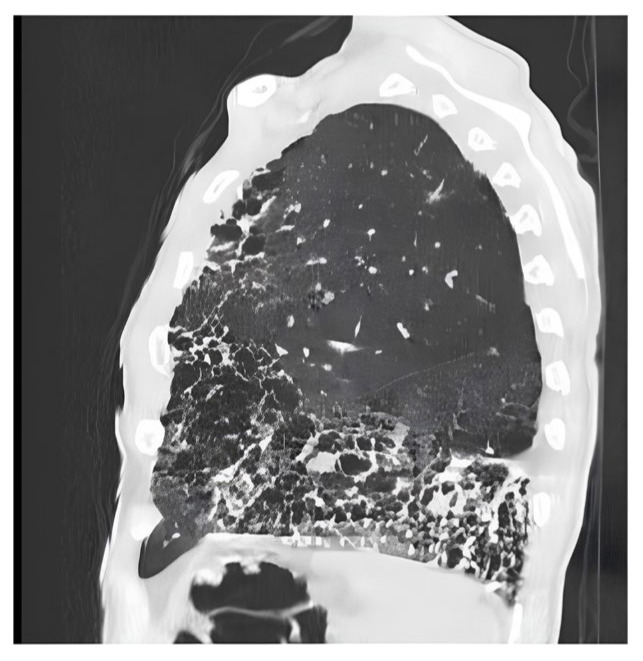
**Sagittal section of a chest CT showing a scan appearance of definite Usual Interstitial Pneumonia (UIP).** The abnormalities show a clear subpleural and basal predominance, with the most severe changes concentrated in the lower lobes of the lung. The hallmark of this pattern, extensive honeycombing, is clearly visible as clustered, thick-walled cystic airspaces arranged in multiple layers. In addition to honeycombing, there are prominent reticular opacities and evidence of traction bronchiectasis, representing the irreversible bronchial and bronchiolar dilatation caused by surrounding fibrous tissue. The combination of these findings, particularly the presence of honeycombing in a subpleural and basal distribution, is highly specific for the UIP pattern.

**Figure 4:**
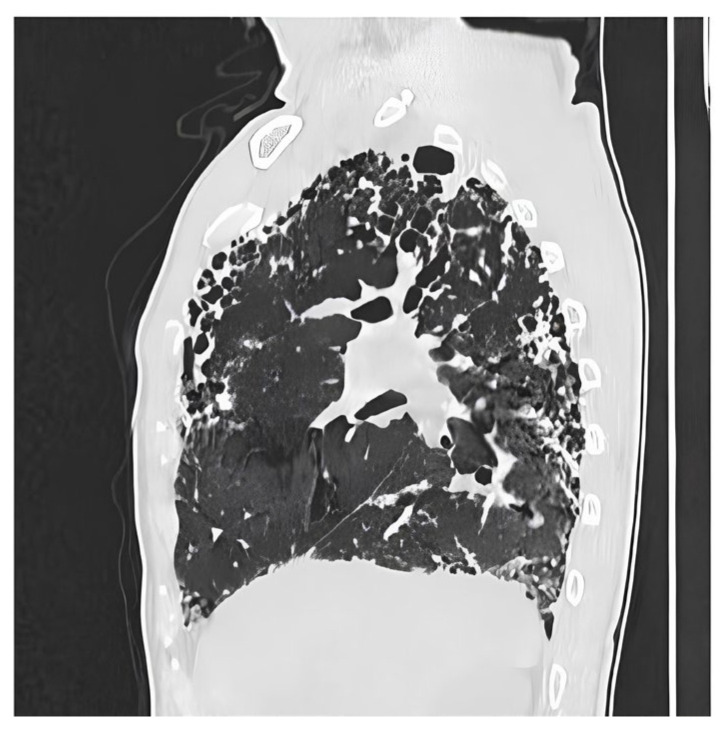
**Sagittal section of a chest CT showing a scan appearance of indeterminate for Usual Interstitial Pneumonia (UIP).** This sagittal HRCT image displays features of advanced pulmonary fibrosis, including prominent reticular opacities, architectural distortion, and clear evidence of honeycombing, visible as multi-layered, clustered cystic airspaces. However, the distribution of these changes is atypical for a classic Usual Interstitial Pneumonia (UIP) pattern. Instead of a distinct basal and subpleural predominance, the fibrotic abnormalities, including the honeycombing, are distributed more uniformly from the upper to the lower parts of the lung.

Definite UIP is defined by the presence of lesions made of honeycomb, bronchiectasis reticulations predominantly located subpleural and basally (Figure 3). Probable UIP displays reticulations and bronchiectasis without honeycombing and exhibits a cranio-caudal gradient. Indeterminate UIP is identified by a diffuse distribution of fibrotic elements without etiological orientation (Figure 4).

Other abnormalities can be visualized on chest CT, namely mediastinal lymphadenopathy in 70% of patients [77], and small areas of calcifications called ossifications (28.5%) [78,79]. Some patients may present signs suggestive of pleuropulmonary fibroelastosis, characterized by irregular pleuropulmonary thickening in the upper and middle lung portions [80].

Biologically, ILDs with UIP patterns may be secondary to connective tissue diseases. This is why the European Respiratory Society (ERS) and the American Thoracic Society (ATS) recommend a series of essential biological examinations to assist in diagnosing IPF, including a complete blood count, antinuclear antibodies (ANA), anti-neutrophil cytoplasmic antibodies (ANCA), rheumatoid factor, anti-cyclic citrullinated peptide (anti-CCP), and the myositis panel (for ATS) [81]. Additional tests may be warranted depending on clinical findings.

In cases of indeterminate UIP, bronchoalveolar lavage BAL can be performed. It shows hypercellularity with increased polynuclear neutrophils (PNN) [82]. Sometimes, there may be an increase in polymorphonuclear eosinophils and lymphocytes but at lower percentages than in cases of eosinophilic lung diseases and sarcoidosis, respectively [81].

The positive diagnosis of IPF relies on the following criteria:

- A scannographic appearance consistent with definite or probable UIP.
- Exclusion of differential diagnoses through comprehensive history-talking and laboratory investigations.

For cases classified as indeterminate UIP, histopathological confirmation is necessary to make the diagnosis [76]. This can be achieved through surgical lung biopsy or, more recently, cryobiopsy. Surgical lung biopsy is the gold standard as it allows for large tissue samples. Ideally, biopsies should be obtained from multiple lobes, targeting pathological areas but avoiding fibrotic zones. The morbidity and mortality associated with this procedure are high (mortality of 1.7% for elective procedures and 17% for non-elective ones) [83]. The risk factors include being of the male gender, having an age greater than 65 years, DLCO < 50%, chronic respiratory failure, pulmonary hypertension, and performing thoracotomy instead of thoracoscopy [84,85,86], and the potential complications include respiratory infections (6.5%), the exacerbation of IPF (6.4%), prolonged air leaks (5.9%), and bleeding (0.8%) [81,86,87]. Transbronchial cryobiopsy is a relatively recent technique that facilitates biopsies via rigid bronchoscopy using a flexible cryoprobe. Its advantages are that it is a less invasive technique than surgical biopsy and results in a higher yield than transbronchial biopsy (74 to 98%) [88].

Overall, the diagnosis of IPF should be established at a multidisciplinary meeting involving pulmonologists, radiologists, rheumatologists, and pathologists. Collaborative discussions enhance diagnostic certainty, determine the necessity for lung biopsy, and discuss therapeutic management.

Recent studies have identified scan abnormalities that can be detected years prior to clinical symptoms, termed preclinical interstitial lung abnormalities (ILA). These abnormalities have become increasingly common due to the increasing demand for chest CT for lung cancer screening and other indications. In patients with ILA, the risk of progression differs, ranging from 20% in 2 years to 50% in 4 to 6 years [89]. The questions that arise are as follows: Should these patients be monitored using cheIf scans? If so at what rate? And would it be beneficial to start antifibrotic treatment at this early stage? All these inquiries remain unresolved and necessitate further research to establish appropriate guidelines. Currently, it is advisable to monitor patients with risk factors for developing IPF or those with a family member with any fibrotic ILD [89].

## 6. Biomarkers: [90,91,92,93,94,95,96,97]

Idiopathic Pulmonary Fibrosis is a heterogeneous disorder in which a tiered biomarker strategy (spanning genetic risk, epithelial injury, immune activation, and ECM remodeling) can improve diagnosis, prognostication, and monitoring when interpreted alongside clinical anchors.

At the genetic level, the MUC5B promoter variant (germline risk variant from blood/saliva DNA) denotes the strongest common susceptibility and, paradoxically, a survival advantage; in contrast, rare variants in telomere-maintenance genes (TERT, TERC, RTEL1, and PARN; germline DNA) and short leukocyte telomere length (peripheral blood) identify more aggressive phenotypes and inform counseling.

Building on this substrate, circulating markers of epithelial injury provide the most actionable signals in routine care: KL-6 (a mucin-type glycoprotein measured in serum/plasma; BALF in research) reflects epithelial activation/regeneration and correlates with disease extent, exacerbations, and mortality; surfactant proteins—particularly SP-D and, to a lesser extent, SP-A (surfactant-associated collectins in serum/plasma; BALF in research)—index type II alveolar epithelial dysfunction and predict functional decline; and MMP-7 (a serum matrix metalloproteinase) robustly captures epithelial–mesenchymal crosstalk and ECM turnover, aligning with HRCT fibrosis burden, FVC/DLCO decline, and mortality, whereas MMP-1 offers a weaker adjunct signal. Complementing epithelial stress, dynamic ECM metrics—collagen formation and degradation neo-epitopes such as PRO-C3, PRO-C6, and C1M/C3M (serum), together with periostin and osteopontin (matricellular proteins in serum/tissue) and LOXL2 (an ECM cross-linking enzyme in serum/tissue)—provide near “real-time” readouts of fibrogenesis and are promising for treatment monitoring, even if the therapeutic targeting of LOXL2 itself has not improved outcomes.

In parallel, immune–inflammatory mediators add prognostic nuance: CCL18 (a macrophage-derived chemokine in serum/plasma) and YKL-40 (a chitinase-like glycoprotein in serum/plasma) repeatedly associate with progression and mortality, and they enhance risk discrimination when integrated with epithelial and ECM markers. Additional biological layers further refine risk: circulating fibrocytes (CD45+Col1+; peripheral blood) track with severe disease and poor survival; microRNAs (e.g., miR-21, let-7d, miR-29 in plasma/tissue) map to fibrotic networks; and TGF-β1 (a canonical profibrotic cytokine in serum/BALF/tissue) serves chiefly as a mechanistic pharmacodynamic marker given assay limitations.

Practically, multiparametric models that combine serum proteins—notably the pragmatic triad of KL-6, SP-D, and MMP-7—with genetic markers, leukocyte telomere length, quantitative HRCT features, and physiologic trajectories (FVC and DLCO) consistently outperform single-analyte strategies for forecasting functional decline, hospitalization, and mortality. Consequently, most markers are best deployed from serum/plasma (with BALF reserved for research phenotyping and germline DNA for genetic testing), and their integrated use provides a coherent, clinically applicable framework for precision risk stratification and longitudinal monitoring in IPF (Table 2).

## 7. Treatment

The treatment of IPF has evolved significantly over recent decades, from the combination of corticosteroids, azathioprine, and N acetyl cysteine, which was the gold standard in the 2000s and is no longer recommended today, to antifibrotic therapies (Nintedanib and Pirfenidone) which have revolutionized the course of the disease and are the treatment recommended nowadays by the different guidelines (American Thoracic Society, European Respiratory Society, Japanese Respiratory Society, and Latin American Thoracic Association (ALAT)) [10].

The management of IPF is multifaceted, focusing on both pharmacological and non-pharmacological interventions.

### 7.1. Pharmalogical Treatment

#### 7.1.1. Approved Antifibrotic Therapies: (Table 3)

##### Nintedanib

Nintedanib is an inhibitor of tyrosine kinase receptors: platelet-derived growth factor (PDGF), fibroblast growth factor (FGF), and vascular endothelial growth factor (VEGF) receptors. This mechanism inhibits the proliferation and migration of fibroblasts, as well as the differentiation of fibroblasts into myofibroblasts [1,2,9,10,98]. Nintedanib received marketing approval from the European Medicine Agency in 2015 and from the U.S Food and Drug Administration (FDA) in 2014.

**Table 3:** Pivotal randomized controlled trials (RCTs) for approved IPF therapies.

Trial Acronym	Drug	Study Design	Primary Endpoint	Key Results
**CAPACITY (1 and 2)**	Pirfenidone	Phase 3, randomized, double-blind, and placebo-controlled (72 weeks)	Change from baseline in percent predicted FVC.	In CAPACITY 2, Pirfenidone significantly reduced the decline in FVC. Pooled data showed a reduction in disease progression.
**ASCEND**	Pirfenidone	Phase 3, randomized, double-blind, and placebo-controlled (52 weeks)	Change from baseline in percent predicted FVC.	Confirmed the findings of CAPACITY, showing a significant reduction in FVC decline. Pooled analysis with CAPACITY demonstrated a reduction in all-cause mortality.
**TOMORROW**	Nintedanib	Phase 2, randomized, double-blind, and placebo-controlled (12 months)	Rate of decline in FVC.	Nintedanib (150 mg twice daily) significantly reduced the rate of FVC decline by ~68% and lowered the incidence of acute exacerbations.
**INPULSIS (1 and 2)**	Nintedanib	Phase 3, randomized, double-blind, and placebo-controlled (52 weeks)	Annual rate of decline in FVC (mL/year).	Both trials met the primary endpoint, showing that Nintedanib slowed FVC decline by ~50% compared to placebo.

Its efficacy has been validated through multiple studies, the most important of which is the TOMORROW study, which included 432 IPF patients treated with Nintedanib for 12 months. This study demonstrated a statistically significant difference between the patients who received the treatment versus those who received the placebo, with a 68.4% reduction in FVC decline, a significant improvement in SGRQ, as well as a reduction in the incidence of exacerbations [99]. The subsequent INPULSIS 1 and 2 trials corroborated these findings. In addition, they showed that patients receiving Nintedanib had a lower all-cause mortality risk than the placebo group [100]. While these studies were carried out on patients with mild respiratory impairment (FVC > 50%), an analysis carried out by L. Rechldi et al. demonstrated the effectiveness and safety of Nintedanib even in patients with a more impaired respiratory function, with a DLCO ≤ 35% [101].

Like any therapy, Nintedanib has side effects that are most often benign such as diarrhea, nausea, anorexia, weight loss, and liver toxicity, with very rare cases of bleeding (epistaxis) [1,99,100,102]. These adverse effects are manageable through a symptomatic treatment or dose adjustment. Therefore, liver function tests are requested before initiating this treatment, with monitoring every month for 6 months, and thereafter every 3 months [1].

##### Pirfenidone

Pirfenidone is a synthetic derivative of 5 methyl 1 phenyl 2 (1H) pyridone, exerting an antifibrotic effect by inhibiting the production of growth factors including TGFβ and the production of collagen. It also reduces the proliferation of fibroblasts and inhibits inflammatory cytokines including TNFα while providing antioxidant effects [2,98,103].

Several studies have evaluated the effectiveness and safety of Pirfenidone. The most recognized are CAPACITY 1 and 2 (studies spread over 72 weeks) [104] and ASCEND (52 weeks) [105]. These studies indicate that Pirfenidone reduces the relative risk of death by 48%, slows the disease progression, reduces the FVC decline, and decreases the relative risk of hospitalization by 48% [106,107,108].

Like Nintedanib, Pirfenidone has side effects that are most often mild and manageable, including digestive disorders (nausea and diarrhea), skin rash, photosensitivity, and hepatotoxicity, and require monitoring of liver function tests identical to those carried out in patients taking Nintedanib [8,10,98].

Nintedanib and Pirfenidone are the two pharmacological agents recommended by guidelines for the treatment of IPF. However, several pertinent questions remain unresolved:

- When should treatment be initiated?
- Which antifibrotic agent should be selected?
- Is there a rationale for combining both treatments?
- At what point should treatment be deemed ineffective, necessitating cessation?

Regarding the optimal timing to initiate treatment, studies have shown that only 60% of patients diagnosed with IPF in Europe and the United States receive antifibrotic treatment [109,110]. This underutilization can be attributed to several factors, including misunderstanding the nature of the disease, problems of accessibility to treatment, and the conviction among some clinicians that patients should be better monitored as long as respiratory function is preserved. On the contrary, it is erroneous that treatment is unwarranted once respiratory function is very impaired with a stage of chronic respiratory failure. Several studies have shown the benefit of early starting of antifibrotic therapy, regardless of the respiratory function impairment, affirming that earlier intervention is advantageous [1,9].

Concerning the choice of treatment, no comparative studies have been conducted. Both treatments have shown their effectiveness in clinical practice, suggesting equivalent therapeutic potentials. The choice of one drug over the other could be made based on the patient’s risk factors, for example, if a patient presents with bleeding risk factors, it would be better to avoid Nintedanib. Conversely, if the patient has skin problems, Pirfenidone should be avoided and Nintedanib should be preferred [111].

Some researchers have looked into the possibility of the potential benefits of combining the two antifibrotic drugs, given that their mechanisms of action are different. An Italian study involved 105 patients who had received Nintedanib 150 mg twice a day for 5 weeks and randomized these patients into two groups: one continued only with Nintedanib while the second received Pirfenidone in addition, at a dose of 801 mg three times a day for 12 months. The results indicated a less significant decline in FVC in the arm of the combination of the two treatments compared to Nintedanib alone, with an identical safety profile (side effects). While these findings suggest the effectiveness and safety of the combination of the two treatments, the very small number of patients as well as the relatively short duration of follow ups preclude definitive conclusions. Larger studies with extended follow-up periods would be desirable before considering this possibility [112].

Currently, the recommendations hardly specify the duration of treatment and the criteria for discontinuation. Ceasing treatment seems logical if major side effects or significant progression of the disease occurs. In terms of the side effects, the majority of patients experience only minor effects that are easily managed by symptomatic treatments or reduction in therapeutic doses. However, the progression of the disease would justify treatment cessation. A subgroup analyzed in the CAPACITY and ASCEND studies showed that patients who progressed on treatment (decline in FVC ≥ 10% in 6 months) and who continued Pirfenidone had a lower risk of subsequent reduction in FVC and death compared to those who stopped it. Also, the INPULSIS-ON study concluded that the effectiveness of Nintedanib continues for at least 3 years [113,114]. A recently published American study indicates that IPF patients with the two TOLLIP rs5743890 and TGFβ rs1800470 polymorphisms demonstrate improved survival with prolonged Pirfenidone treatment [115].

##### Nerandomilast

Nerandomilast, a novel selective phosphodiesterase 4B (PDE4B) inhibitor, received FDA approval on 7 October 2025, for the treatment of Idiopathic Pulmonary Fibrosis. This approval marks a significant advancement in IPF therapy, introducing a new class of oral medication for this progressive and fatal lung disease. The approval was based on the positive results from the pivotal Phase III FIBRONEER-IPF trial, a randomized, double-blind, placebo-controlled study involving patients with IPF.

The primary endpoint of the trial was the annual rate of decline in forced vital capacity (FVC). Patients treated with nerandomilast demonstrated a statistically significant reduction in the rate of FVC decline compared to those receiving a placebo. For instance, the adjusted annual rate of decline in FVC was −85.5 mL with nerandomilast versus −179.9 mL with placebo, representing a 52.5% relative reduction.

The mechanism of action of nerandomilast involves the inhibition of PDE4B, which leads to an increase in intracellular cyclic adenosine monophosphate (cAMP). This increase modulates the activity of various pro-inflammatory and profibrotic pathways implicated in the pathogenesis of IPF. Preclinical studies have shown that nerandomilast effectively reduces lung fibrosis and inflammation by downregulating key fibrotic markers and pathways. Its safety profile was found to be generally acceptable; the most common adverse events reported in the clinical trials were gastrointestinal in nature, including diarrhea and nausea, which were typically mild to moderate in severity [116,117,118,119].

#### 7.1.2. Investigational Drugs with Promising Results

Recent advancements in the understanding of the pathophysiology of IPF, along with the elucidation of growth factor and cytokine pathways, have led to the development of several molecules currently undergoing clinical trials to investigate their efficacy in the treatment of IPF [120,121,122,123]. There are even teams who have looked into the interest of stem cells delivered endobronchially or bone marrow cells delivered intravenously in the treatment of IPF, with promising results, but more broad-spectrum studies are required to confirm them [124,125,126]. Other scientists have been interested in the induction of tolerance to the autoantigens responsible for the aberrant immune responses (phase I study with encouraging results (stability of FVC)) [127].

Among these therapeutic agents, one molecule stands out and emerges as a leading candidate poised to enhance the IPF’s therapeutic arsenal: Pamrevlumab. It is a monoclonal antibody targeting CTGF (connective tissue growth factor). CTGF activates the growth factor pathway such as TGFβ and therefore stimulates the synthesis of the extracellular matrix and the dysregulation of fibrous tissue [1,2,3,4,5,6,7,8,9,10,11,12,13,14,15,16,17,18,19,20,21,22,23,24,25,26,27,28,29,30,31,32,33,34,35,36,37,38,39,40,41,42,43,44,45,46,47,48,49,50,51,52,53,54,55,56,57,58,59,60,61,62,63,64,65,66,67,68,69,70,71,72,73,74,75,76,77,78,79,80,81,82,83,84,85,86,87,88,89,90,91,92,93,94,95,96,97,98,99,100,101,102,103,104,105,106,107,108,109,110,111,112,113,114,115,116,117,118,119,120]. The PRAISE study carried out on 103 IPF patients randomized into two groups: 53 receiving Pamrevlumab 30 mg/kg IV every 3 weeks for 48 months and the other group a placebo, demonstrated that the treatment slowed the FVC decline by 60.3% with an improvement in the SGQL quality of life score [128]. Phase III trials have begun, with very encouraging results. There are even metanalyses which have already been interested in comparing the effects of the three antifibrotic drugs, Nintedanib, Pirfenidone, and Pamrevlumab, with the majority concluding in a similar and close effect [42], through a study by Di Mortino et al. highlighted the greater efficacy of Pamrevlumab compared to the other antifibrotic drugs in terms of reducing the FVC decline [111].

One other promising agent is bexotegrast (PLN-74809), a dual-selective inhibitor of the αvβ6 and αvβ1 integrins, which are key mediators in the activation of TGF-β, a central profibrotic cytokine. In a phase 2a trial involving patients with IPF, bexotegrast was found to be well-tolerated and demonstrated a significant reduction in the TGF-β1 levels in bronchoalveolar lavage fluid after 12 weeks of treatment. This was accompanied by a slower decline in forced vital capacity (FVC) in the treatment groups compared to the placebo, suggesting a potential for modifying disease progression and promoting lung remodeling [129,130].

Another novel approach involves targeting the lysophosphatidic acid (LPA) pathway, which is implicated in fibroblast recruitment and activation. Admilparant, a potent antagonist of the lysophosphatidic acid receptor 1 (LPA1), was evaluated in a phase 2 trial in patients with IPF and other progressive pulmonary fibrotic diseases. The study showed a significantly delayed disease progression, as measured by the rate of FVC decline over 26 weeks, compared to the placebo. The safety profile was acceptable, marking it as a potential future therapy for a broad range of fibrotic lung diseases [131].

Rentosertib, a small molecule inhibitor of TRAF2- and NCK-interacting kinase (TNIK) discovered through artificial intelligence, has also shown potential. TNIK is involved in the Wnt signaling pathway, which plays a role in fibrosis. A phase 2a clinical trial demonstrated that rentosertib was safe and well-tolerated in patients with IPF. Notably, patients receiving the highest dose of rentosertib experienced a significant improvement in FVC at 12 weeks, suggesting a beneficial effect on lung function [132].

Furthermore, efforts are being made to improve the tolerability of existing treatments. An inhaled formulation of Pirfenidone (AP01) has been developed to deliver the drug directly to the lungs, thereby minimizing systemic side effects. A recent study showed that inhaled Pirfenidone had significantly fewer gastrointestinal and skin-related adverse events compared to the oral formulation. While the primary endpoint of FVC change was not met, the data suggested a potential for stabilizing lung function with a much-improved safety profile, which could be advantageous for patients who cannot tolerate oral Pirfenidone [133].

Recently, in 2020, a Swiss team demonstrated that azithromycin exhibits a significant antifibrotic effect regarding the secretion of collagen and fibronectin, as well as the differentiation of myofibroblasts in IPF patients compared to controls. Furthermore, it improved the pro-apoptotic effect on the fibroblasts of IPF patients. Nonetheless, larger studies are required before drawing conclusions [134].

As for N acteyl cysteine NAC, a drug widely prescribed in the 2000s in combination with corticosteroid therapy and azathioprine, several studies have investigated the efficacy of adding NAC to Pirfenidone in IPF patients. The PANORAMA and the Japanese study by S. Sakomoto showed no benefits of taking NAC [135,136], while the PANTHER study suggested that NAC would be beneficial in a population of patients with a polymorphism in the gene encoding TOLLIP rs3750920 with genotype T/T [29].

#### 7.1.3. The Shift Toward Combination and Personalized Therapies

The future of IPF management is moving toward more sophisticated strategies. The heterogeneity of disease progression suggests that the “one-size-fits-all” approach has its limits. The current trend is to explore combination therapies, pairing an approved antifibrotic with an investigational agent that has a complementary mechanism of action (e.g., one that is anti-inflammatory or targets a different fibrotic pathway). Furthermore, the use of biomarkers, such as PRO-C3, to stratify patients and predict their response to treatment is an active area of research. This precision medicine approach aims to tailor treatment to each patient’s molecular and clinical profile, with the goal of improving efficacy and minimizing side effects.

### 7.2. Non-Pharmacological Treatment

The non-pharmacological management of IPF is as important as medication, as it allows better adaptation to the disease and enhance the quality of life for patients suffering from this severe chronic condition.

#### 7.2.1. Oxygen Therapy

The various guidelines recommend the use of long-term oxygen therapy in IPF patients with chronic respiratory failure. It is even preferable to introduce ambulatory oxygen therapy for patients who desaturate during physical activity, since it improves dyspnea and exercise capacity [2,4,8].

#### 7.2.2. Pulmonary Rehabilitation

Pulmonary rehabilitation is a structured exercise program established in patients with chronic pulmonary pathology, aimed at optimizing exercise capacity and quality of life. The objectives are to improve the physical and psychological state of the patient and to increase adherence to treatment. Several studies have evaluated the impact of 12 to 16 weeks of pulmonary rehabilitation on patients with IPF and resulted in an improvement in the distance covered in the 6 min walk test, dyspnea, and quality of life [4,137,138]. A Portuguese case–control study involving 32 IPF patients and 15 controls, who benefited from a rehabilitation program over a period of 12 weeks, indicates additional benefits, including improvement in pulmonary function (assessed by FVC), dyspnea (mMRC scale), and muscle strength (30 s chair rise test) [139].

All these elements support the early initiation of pulmonary rehabilitation in IPF patients. Unfortunately, the effect of this rehabilitation does not persist beyond 6 months [140].

#### 7.2.3. Lung Transplantation

Recent statistics indicate that over 4600 lung transplants are performed worldwide each year, with approximately half of which are for interstitial lung diseases [141]. The natural progression of IPF is chronic respiratory failure, PH, and death. Therefore, lung transplantation remains a viable option. It is imperative to refer these patients to a specialized center as early as possible in order to be included in the lists and to carry out the genetic study to detect patients with a TERT mutation carrying a high risk of post-transplant mortality [8].

#### 7.2.4. Vaccination

IPF patients are elderly individuals with chronic pulmonary conditions, who are exposed to a high risk of death if they develop a pulmonary infection [142,143]. Therefore, it is recommended for all IPF patients to receive the annual influenza vaccination and the pneumococcal vaccination [8].

### 7.3. Treatment of Symptoms

Antifibrotic treatments are known to decelerate the decline in respiratory function; however, their impact on symptoms is minimal. Therefore, it is imperative to pay attention to the symptoms the patient reports and treat them.

#### 7.3.1. Management of Cough

A chronic cough is a common and disabling symptom in patients with IPF, significantly impacting their quality of life. It affects 20 to 80% of IPF patients [121,144].

Management relies on non-pharmacological approaches, such as speech therapy, and low-level pharmacological treatments like low-dose thalidomide or neuromodulators, such as gabapentin [145].

#### 7.3.2. Management of Dyspnea [8,144]

Dyspnea is the most disabling symptom of IPF. Its management is multimodal and includes pulmonary rehabilitation, oxygen therapy to correct hypoxemia, and the use of low-dose opioids to relieve the sensation of breathlessness in advanced stages. These interventions aim to improve exercise capacity and quality of life.

## 8. Comorbidities and Complications

### 8.1. Comorbidities

Comorbidities are defined by an event with a frequency higher than expected, presenting an etiopathogenic link with the disease and influencing on the pathophysiology, diagnosis, severity, and prognosis. It is essential to distinguish between comorbidity and complication [146].

IPF is frequently associated with numerous comorbidities. According to a recent study, 60% of IPF patients present with one to three comorbidities, while only 10% have no comorbidities.

These comorbidities can be categorized into two groups: respiratory and extra respiratory.

#### 8.1.1. Respiratory Comorbidities

➢ Combined Pulmonary Fibrosis and Emphysema (CPFE) syndrome

CPFE, first described by Vincent Cottin in 2005, has a prevalence ranging from 8 to 51%, with a median accuracy in one third of patients with IPF. It is characterized by increased dyspnea with oxygen desaturation, and a reduced DLCO contrasting with preserved lung volumes. CPFE diagnosis is established via chest CT, which reveals emphysema in the upper lobes and fibrosis in the lower lobes. The primary complications of this syndrome are pulmonary hypertension and bronchogenic cancer, which worsens its prognosis, with a median survival of 25 months. While treatment protocols are not standardized, a comprehensive approach involving antifibrotic therapy for fibrosis, smoking cessation, and bronchodilator treatment for the emphysematous component is generally warranted [8,146,147,148].

➢ Bronchopulmonary cancer

IPF patients exhibit a fivefold increased risk of developing bronchogenic cancer compared to the general population. The cumulative incidence of cancer risk increases over time with approximately 3.3% at 1 year post-diagnosis, 15.4% at 5 years, and 54.7% at 10 years [148]. Clinically, these patients do not present any particular symptoms (hemoptysis or weight loss) and, radiologically, the diagnosis is often complicated, given that the tumor lesions are often peripheral and located in the lower lobes and therefore overlap with the fibrosis lesions. The predominant histological type is squamous cell carcinoma [146]. The treatment must be fully discussed in a multidisciplinary meeting given that most therapies are poorly tolerated [148]. As for antifibrotic treatment, it is recommended to keep it because of its prophylactic role in reducing post-operative mortality as well as its synergistic action with anti-cancer treatments [8].

➢ Obstructive Sleep Apnea Syndrome OSA

Highly prevalent in IPF patients (up to 88%), OSA may contribute to lung injury through mechanical stress and intermittent hypoxia [63]. It should be systematically screened for [63]. The management of OSA-IPF is the same as that for OSA without IPF.

#### 8.1.2. Extra Respiratory Comorbidities

➢ Gastroesophageal reflux (GERD)

As the most common comorbidity (up to 80% of patients) [63,148], GERD is thought to contribute to lung injury via microaspiration. However, the causal link is complex, and current guidelines recommend treating only symptomatic patients with anti-acid therapy [71,149].

➢ Other comorbidities

Cardiovascular diseases (coronary heart disease and hypertension), diabetes, hematological abnormalities, sarcopenia, malnutrition, anxiety, and depression are also frequently observed and require management [150,151,152].

### 8.2. Complications

IPF is further challenged by the high incidence of associated complications that require distinct therapeutic interventions and represent a major source of morbidity, independent of the underlying fibrotic progression. The most important complications are as follows:

#### 8.2.1. Acute Exacerbation of IPF

An acute exacerbation of Idiopathic Pulmonary Fibrosis (AE-IPF) represents a sudden and clinically significant respiratory deterioration, which is formally diagnosed based on a set of refined criteria (2016). These require a prior or concurrent diagnosis of IPF in a patient presenting with an acute worsening of dyspnea within a one-month timeframe, confirmed by new bilateral ground-glass opacities and/or consolidations on high-resolution computed tomography (HRCT) that are superimposed on the underlying fibrotic pattern. A critical component of this diagnosis is the systematic exclusion of alternative etiologies, such as cardiac failure, fluid overload, or pulmonary embolism [153,154]. Its annual incidence is estimated to be between 5% and 20% [155,156] and the prognosis remains poor (mortality rate up to 80%), with a median survival of 3 to 4 months [101,157,158,159,160,161]. The most significant predictors include measures of impaired pulmonary function (FVC and DLCO), as well as the presence of pulmonary hypertension and elevated serum levels of KL-6 [162]. Other risk factors include a history of exacerbations, cardiac comorbidity, being of a young age, and having a high BMI [154,157,163], with studies suggesting the role of viral infections and gastric microaspiration in the development of this situation [70,164,165,166]. The therapeutic approach remains a subject of considerable debate. Systemic corticosteroids are widely recommended as a first-line therapy, though their efficacy is uncertain. The addition of immunosuppressive agents is now generally avoided due to evidence of increased mortality, whereas macrolides have shown encouraging results for their potential immunomodulatory effects [8,167,168,169,170]. Core management relies on supportive care, including supplemental oxygen and non-invasive ventilation, with broad-spectrum antibiotics typically administered empirically. The use of antifibrotic therapies have been proven to substantially lower the relative risk of a first acute exacerbation (RR of 0.63) [69,171].

#### 8.2.2. Pulmonary Hypertension PH

A common complication (8 to 15% of patients at the time of diagnosis) [8,148], PH prevalence increases as IPF progresses, reaching up to 86% in advanced stages % [147]. Clinically, PH is suspected in patients presenting disproportionate dyspnea, desaturation on the 6 min walk test, reduction in DLCO, dilated right heart cavities with increased isovolumetric pulmonary artery velocity (IT Vmax) on transthoracic echocardiography, or an enlarged pulmonary artery diameter. Diagnosis is confirmed by right heart catheterization. Treatment is focused on correcting hypoxia with oxygen therapy, as specific vasodilator therapies have not proven effective [172,173,174].

## 9. Conclusions

In the past decade, we have witnessed a rise in the prevalence of IPF, which is attributed to better awareness of its symptoms by physicians and the increase in demand for chest scans. IPF will no longer be a rare disease in few years. We made progress in terms of understanding its genetic mechanisms, which will enable us to improve its management. Current research efforts are focused on improving methods of earlier diagnosis, subphenotyping and the prognostication of patients with genetic biomarkers. We aspire to discover novel treatments that could change the disease trajectory by stabilizing or reversing the fibrotic process. Until then, the current antifibrotics, which modify the course of IPF, diagnostics, and the treatment of comorbidities can contribute to improved quality of life and extended life expectancy for patients, despite the unpredictable nature of the disease’s outcome.

## Figures and Tables

**Table 1:** Comprehensive table of genetic variants associated with Idiopathic Pulmonary Fibrosis (IPF).

Gene	Variant Type	Associated Pathway/Function	Key Findings in IPF
**Common Variants (Allele Frequency > 1%)**			
*MUC5B* [17,18,19,20,21,22,23,24,25]	Common (rs35705950)	Mucin production; host defense	The strongest genetic risk factor for both familial and sporadic IPF. The T allele leads to MUC5B overexpression, mucociliary dysfunction, and ER stress. Paradoxically, carriers of the T allele are associated with better survival outcomes.
*TOLLIP* [26,27,28,29]	Common (rs5743890, rs3750920)	Innate immunity (Toll-like receptor signaling)	Modulates inflammatory responses. The minor allele of rs5743890 is linked to poorer survival and faster disease progression. The rs3750920 variant shows a significant interaction with N-acetylcysteine (NAC) therapy.
*FAM13A* [30,31,32,33]	Common	Wnt signaling	Identified in GWAS as a susceptibility locus.
*DSP* [30,31,32,33]	Common	Cell adhesion	Identified in GWAS as a susceptibility locus.
*OBFC1* [30,31,32,33]	Common	DNA repair; telomere maintenance	Identified in GWAS as a susceptibility locus.
*ATP11A* [30,31,32,33]	Common	Unknown in IPF context	Identified in GWAS as a susceptibility locus.
*DPP9* [30,31,32,33]	Common	Inflammation	Identified in GWAS as a susceptibility locus.
*SPPL2C* [30,31,32,33]	Common	Unknown in IPF context	Identified in GWAS as a susceptibility locus.
*PKN2* [30,31,32,33]	Common	Unknown in IPF context	A variant has been associated with disease progression, potentially revealing a new biological mechanism.
*GPR157*, *DNAJB4*/*GIPC2*, *RAPGEF2*, *FKBP5*, *RP11286H14.4*, *PSKH1*, *FUT6* [30,31,32,33]	Common	Various	Identified as novel susceptibility loci in a large multi-ancestry meta-analysis.
**Rare Variants (Allele Frequency < 1%)**			
Telomere-Related Genes			
*TERT*, *TERC* [34,35,36,37,38,39,40,41,42,43,44]	Rare	Telomere maintenance (telomerase components)	Monoallelic mutations cause telomere shortening, leading to premature cellular senescence and impaired epithelial repair. Found in a significant portion of familial IPF cases.
*RTEL1*, *PARN* [45]	Rare	Telomere maintenance	Mutations also lead to telomere shortening and are associated with familial IPF. RTEL1 is a helicase; PARN is involved in TERC RNA processing.
*DKC1*, *ZCCHC8*, *NAF1* [46,47]	Rare	Telomerase biogenesis	Mutations in these genes, which are essential for the assembly and function of telomerase, have been identified in IPF.
*TINF2*, *ACD* [48,49]	Rare	Telomere integrity (Shelterin complex)	Heterozygous mutations in these genes, which encode proteins that protect telomeres, have been documented in IPF.
Surfactant-Related Genes			
*SFTPA1*, *SFTPA2* [50,51,52,53,54,55]	Rare	Surfactant protein A production and function	Mutations (e.g., F198S, G231V in SFTPA2) are located in the carbohydrate recognition domain, leading to misfolded proteins, ER stress, and apoptosis in alveolar epithelial cells. Associated with early-onset fibrosis and lung cancer risk.
*SFTPC* [56,57,58,59]	Rare	Surfactant protein C production and function	Mutations, often in the BRICHOS domain, cause misfolded SP-C to accumulate in the ER, inducing ER stress. Predisposes to a wide range of fibrotic lung diseases.
*SFTPB*	Rare	Surfactant protein B production and function	Pathogenic variants have been associated with IPF.
*ABCA3* [60,61,62]	Rare	Surfactant lipid transport	Mutations impair the function of this transporter in lamellar bodies, disrupting surfactant synthesis and metabolism and contributing to epithelial cell injury.
*NKX2.1* [60]	Rare	Lung development; surfactant protein transcription	Pathogenic variants have been associated with IPF.

**Table 2:** Selected biomarkers in Idiopathic Pulmonary Fibrosis.

Biomarker	Type	Source	Clinical Applicability and Significance
KL-6 (MUC1)	Mucin-type glycoprotein (epithelial injury marker)	Serum/plasma; BALF (research)	Aids differential diagnosis among ILDs; higher levels associate with disease extent, progression, acute exacerbations, and mortality; and they are useful for longitudinal monitoring (widely used in Japan).
Surfactant Protein D (SP-D)	Surfactant-associated collectin	Serum/plasma; BALF (research)	Reflects alveolar epithelial injury; elevated in IPF; predicts FVC decline and mortality; and useful for disease monitoring.
Surfactant Protein A (SP-A)	Surfactant-associated collectin	Serum/plasma; BALF (research)	Elevated in IPF; supports diagnosis/monitoring; prognostic value generally weaker than SP-D.
MMP-7	Matrix metalloproteinase (ECM remodeling)	Serum/plasma	One of the most consistently validated prognostic biomarkers; predicts progression and mortality; correlates with HRCT fibrosis extent and FVC decline; and often used in multi-marker panels.
MMP-1	Matrix metalloproteinase	Serum/plasma	Elevated in IPF; associated with disease activity and fibrosis remodeling, though its prognostic value is less robust than MMP-7.
CCL18 (PARC)	Chemokine (macrophage-derived)	Serum/plasma	Higher baseline concentrations predict mortality and acute exacerbations; tracks disease activity; independent prognostic signal in several cohorts.
YKL-40 (CHI3L1)	Chitinase-like glycoprotein	Serum/plasma	Associated with fibrosis burden, decline in lung function, and mortality; reflects epithelial injury/repair and macrophage activation.
Periostin (POSTN)	Matricellular ECM protein	Serum/plasma; lung tissue	Elevated in progressive IPF; associated with fibrogenic activity and worse outcomes; and potential tool for risk stratification and treatment monitoring.
Osteopontin (SPP1)	Cytokine/matricellular protein	Serum/BALF; lung tissue	Upregulated in IPF; correlates with disease severity and progression; and pathway target under investigation.
LOXL2	ECM cross-linking enzyme	Serum; lung tissue	Marker of active matrix remodeling; higher levels associated with severity and progression; and therapeutic targeting to date has not improved outcomes (utility mainly as disease activity marker).
MUC5B promoter variant (rs35705950)	Genetic risk variant	Germline DNA (blood/saliva)	Strongest common genetic risk factor for IPF; paradoxically linked to better survival; and useful for risk stratification and research, not diagnostic alone.
Telomere-related genes (TERT, TERC, PARN, RTEL1)	Genetic variants (telomere maintenance)	Germline DNA; leukocyte telomere length	Mutations and short telomeres are associated with familial/sporadic IPF, earlier onset, worse outcomes, and transplant complications; they inform counseling and management.
Leukocyte telomere length	Genomic/aging biomarker	Peripheral blood leukocytes	Shorter telomeres predict faster progression, poorer survival, and toxicity risk with some therapies; complements genetic testing.
Circulating fibrocytes	Cellular biomarker (CD45+Col1+)	Peripheral blood	Elevated percentages predict worse survival and severe disease; potential marker of fibrogenic activity (research/selected centers).
ECM neo-epitopes (e.g., PRO-C3, PRO-C6; C1M/C3M)	Collagen turnover fragments	Serum	Reflect active fibrogenesis and ECM turnover; associate with progression and mortality; and promising for treatment monitoring.
microRNAs (e.g., miR-21, let-7d, miR-29)	Non-coding RNAs (regulatory)	Plasma/serum; lung tissue	Dysregulated in IPF; linked to fibrotic pathways and outcomes; and emerging prognostic/theranostic markers (research stage).
TGF-β1	Cytokine (profibrotic)	Serum/BALF; tissue	Central to fibrosis biology; elevated but nonspecific; limited standalone clinical utility; and useful in mechanistic and pharmacodynamic studies.

## Data Availability

No new data were created or analyzed in this study. Data sharing is not applicable to this article.

## Abbreviations

The following abbreviations are used in this manuscript:

AE-IPF	Acute Exacerbation of Idiopathic Pulmonary Fibrosis
ALAT	Latin American Thoracic Association
ANA	Antinuclear Antibody
ANCA	Anti-Neutrophil Cytoplasmic Antibodies
ATP11A	ATPase Phospholipid Transporting 11A
ATS	American Thoracic Society
BAL	Bronchoalveolar Lavage
CPFE	Combined Pulmonary Fibrosis and Emphysema
CTGF	Connective Tissue Growth Factor
CXCL	Chemokine (C-X-C motif) Ligand
DLCO	Diffusing Capacity of the Lungs for Carbon Monoxide
DPP9	Dipeptidyl Peptidase 9
ERS	European Respiratory Society
FGF	Fibroblast Growth Factor
FVC	Forced Vital Capacity
GERD	Gastroesophageal Reflux Disease
HRCT	High-Resolution Computed Tomography
ILA	Interstitial Lung Abnormality
ILD	Interstitial Lung Disease
IPF	Idiopathic Pulmonary Fibrosis
JRS	The Japanese Respiratory Society
KIF15	Kinesin Family Member 15
KL6	Krebs von den Lungen-6
LPA	Lysophosphatidic Acid
LPA1	Lysophosphatidic Acid Receptor 1
MAD1L1	MAD1 Mitotic Arrest Deficient 1 Like 1
MMP	Matrix Metalloproteinase
mMRC	Modified Medical Research Council
MUC5B	Mucin 5B
OSA	Obstructive Sleep Apnea
PDGF	Platelet-Derived Growth Factor
PFT	Pulmonary Function Test
PH	Pulmonary Hypertension
RCT	Randomized Controlled Trial
SFTPA	Surfactant Protein A
SGRQ	St. George’s Respiratory Questionnaire
SP	Surfactant Protein
TERC	Telomerase RNA Component
TERT	Telomerase Reverse Transcriptase
TGF-β	Transforming Growth Factor-Beta
TKI	Tyrosine Kinase Inhibitor
TNIK	TRAF2 and NCK-Interacting Kinase
TNFα	Tumor Necrosis Factor alpha
TOLLIP	Toll Interacting Protein
UIP	Usual Interstitial Pneumonia
VEGF	Vascular Endothelial Growth Factor
